# Mycoplasma‐Induced Rash and Mucositis: Ocular Manifestations, Treatment and Outcomes in a Paediatric Population

**DOI:** 10.1111/jpc.70103

**Published:** 2025-06-05

**Authors:** Daniel Cool, Vivien Nguyen, Susan Zhang, Shiney Seo, Shuan Dai

**Affiliations:** ^1^ Ophthalmology Department Queensland Children's Hospital Brisbane Queensland Australia; ^2^ Faculty of Medicine University of Queensland Australia; ^3^ Menzies Health Institute Queensland Griffith University Casuarina Australia

**Keywords:** community, dermatology, general paediatrics, immunology, infectious diseases

## Abstract

**Background:**

There has been an increase in *Mycoplasma pneumoniae* infections. This can present with extra‐pulmonary manifestations including mycoplasma‐induced rash and mucositis (MIRM). This paper aims to describe the ocular features in MIRM at a paediatric tertiary referral hospital in Queensland, Australia, and review the treatment and outcomes of these patients.

**Methods:**

A case series of 14 patients presenting to the Queensland Children's Hospital was included in this study. Patient demographics, ocular examination findings, and treatment methods were obtained from retrospective chart review.

**Results:**

The mean age was 11.2 years (range 8–15 years) and was mostly male (73%). All patients had conjunctival injection or ulceration. No patients had corneal involvement. One patient underwent amniotic membrane graft and glue tarsorrhaphy for one eye. All others were managed non‐surgically with cautious use of topical steroid, as well as multidisciplinary care. Most patients (80%) achieved full recovery with no symblepharon formation or forniceal shortening.

**Conclusion:**

Conservative management with preservative‐free antibiotics and lubricants should be considered as initial first‐line treatment for patients presenting with MIRM. Topical corticosteroid therapy should be guided by an ophthalmology service. This should be in conjunction with systemic therapy and multidisciplinary care for extra‐ocular complications. This case series suggests a treatment guideline for future patients presenting with possible MIRM. Paediatric and primary care physicians should be aware of ocular features of MIRM to facilitate early referral to and intervention by ophthalmologists to allow for the best outcome.

**Summary**

- *Question*: What are the ocular features in mycoplasma‐induced rash and mucositis?
- *Findings*: The main ocular features found were conjunctival injection, conjunctival epithelial loss, and subconjunctival haemorrhage.
- *Meaning*: Paediatric patients presenting with red eyes after a viral prodrome or with upper respiratory tract symptoms should be investigated for mycoplasma‐induced rash and mucositis with early ophthalmology involvement to optimise treatment and recovery.

## 1 Background

There has been an increased incidence of *Mycoplasma pneumoniae (M. pneumoniae)* infections in Australia by 150% this year to date, based on results at a single private pathology practice alone [1]. Paediatric patients accounted for at least 60% of positive results [1]. Mucocutaneous disease such as Mycoplasma‐induced rash and mucositis (MIRM) can be seen in up to 23% of paediatric patients with *M. pneumoniae*, who are often more systemically unwell and have worse long‐term sequelae. MIRM refers to patients with severe mucositis (e.g., ocular, oral, urogenital) with or without skin involvement and is estimated to occur in 7% of patients with *M. pneumoniae* infection [2] and, although clinical features are similar, can be considered as a separate disease entity to Stevens‐Johnson Syndrome (SJS), toxic epidermal necrolysis syndrome (TENS) or reactive infectious mucocutaneous eruption (RIME) [2, 3].

MIRM was previously described as a subclass of SJS/TEN, rather than a separate disease entity with unique pathophysiology [2, 3, 4]. There is limited existing literature regarding the ocular manifestations and treatment recommendations in MIRM [5, 6, 7, 8, 9, 10]. To date, the largest published case series was of fifteen patients [6]. Our series of fourteen represents the largest Australian cohort.

Ocular features of SJS/TENS and MIRM include conjunctival injection, conjunctival epithelial defects, pseudomembranes, and forniceal shortening [11], eyelid margin necrosis, and keratinisation. Corneal changes may include superficial punctate keratitis, punctate epithelial erosions, ulceration, corneal perforation, and corneal opacification [12, 13]. SJS/TENS mucositis carries a significantly worse visual outcome than MIRM due to severe cicatricial changes [5, 14]. The severity of acute SJS/TENS is the most reliable predictor of chronic eye complications [15]. This is yet to be determined for MIRM, which carries a significantly lower mortality risk than SJS/TENS [16].

Both MIRM and SJS/TENS tend to involve multiple mucosal sites and can cause severe mucositis, even without skin involvement [2, 17, 18], SJS/TENS is usually associated with medication exposure and presents with extensive cutaneous lesions [19, 20]. In contrast, MIRM has variable cutaneous involvement and often presents with severe oral mucositis after a preceding cough [2, 20, 21].

Current treatment guidelines for ocular complications associated with MIRM are varied, with some suggesting amniotic membrane grafting and intensive topical management, similar to patients with SJS/TENS [5, 22], while others have suggested good outcomes with moderate topical treatment without amniotic membrane grafting [23].

This study aims to report on ocular manifestations of MIRM at a tertiary paediatric hospital, focusing on ophthalmic care and outcomes, to provide evidence for potential development of management guidelines in patients with MIRM.

## 2 Methods

### 2.1 Study Design, Population and Settings

This was a retrospective cohort review of paediatric patients with MIRM, who had been referred to the Queensland Children's Hospital ophthalmology department between January 2023 and May 2024. Inclusion criteria were patients under 18 years of age and laboratory confirmed mycoplasma diagnosis by *M. pneumoniae* respiratory PCR or *M. pneumoniae* serum antibody testing. Patients diagnosed with other mucocutaneous conditions such SJS or TENS were excluded. After inclusion and exclusion criterion were applied, 14 patients (28 eyes) were identified. Data was collected from integrated electronic medical records (iEMR). Ethics exemption for this study was granted by the Children's Health Queensland Hospital and Health Service Human Research Ethics Committee (HREC/2024/QCHQ/109296). The Tenets of the Declaration of Helsinki were followed. Individual patient consent was obtained from the parent/guardian for inclusion of clinical photographs.

### 2.2 Study Measures

Demographic information including gender, age at presentation, ethnicity, and locality were obtained from iEMR. Clinical characteristics collected included presenting visual acuity (VA), ophthalmological findings, systemic manifestations, onset of systemic symptoms, laboratory diagnosis, ocular and systemic treatment, onset and resolution of ocular symptoms, and final ophthalmological outcome. Ocular severity was retrospectively categorised based on the largest documented total conjunctival epithelial defect (ED), corneal involvement, and change in visual acuity: mild–moderate for less than 50% total conjunctival involvement; or severe if greater than or equal to 50% total conjunctival involvement with or without cicatricial disease (pseudomembranes, forniceal shortening, symblepharon). Any corneal involvement independent of conjunctival involvement was considered severe. A decrease in visual acuity of more than LogMAR 0.20 from baseline (if known) or from presumed LogMAR 0.00 was considered severe. Lid margin involvement was also a severe finding. Systemic severity was retrospectively categorised as mild–moderate, defined as mucocutaneous involvement without systemic intervention; or severe, which was defined as mucocutaneous involvement requiring systemic intervention such as supplemental oxygen, admission to the intensive care unit (ICU), indwelling catheter (IDC) or nasogastric tube (NGT) insertion.

### 2.3 Data Analyses

The collected data was analysed using IBM SPSS Statistics version 27. Descriptive statistics was generated for population demographics. Univariate analysis was used to determine factors affecting mean days of disease resolution. Confounding variables included frequency of topical treatment (antibiotic, steroid, lubricants), ocular severity, and systemic severity. A two‐sided *p* < 0.05 was considered statistically significant.

## 3 Results

Fourteen patients were identified with ocular involvement of MIRM (Table 1).

**TABLE 1:** Patient demographics.

Age in years: mean (range)	11.2 (8–15)
Gender: number (%)	Male 10 (75%), Female 4 (25%)
Ethnicity: number (%)	
Caucasian	11 (78.6%)
Indian	1 (7.1%)
Other/unknown	2 (14.3%)
Patient location: number (%)	
Brisbane (Southside)	2 (14.3%)
Brisbane (Northside)	2 (14.3%)
Logan	4 (28.6%)
Ipswich	3 (21.4%)
Moreton Bay	1 (7.1%)
Mackay	1 (7.1%)

All patients had preceding respiratory symptoms before the onset of mucositis, at which time 50% of patients had been treated for community‐acquired pneumonia with either doxycycline (*n* = 1), amoxicillin (*n* = 1), doxycycline and amoxicillin (*n* = 1) or azithromycin (*n* = 4). Ocular symptoms followed the onset of systemic/respiratory symptoms by 6.2 ± 3.14 days.

Mycoplasma PCR testing was positive for 69% of patients tested (*n = 13*) and Mycoplasma serology tested on average 8.4 days after onset of systemic symptoms was positive in 92% of tested patients (*n = 1*2). All four patients who tested negative on PCR were positive on serological testing, and one patient who tested negative on serological testing 4 days after onset of systemic symptoms was positive on PCR testing.

Thirteen (92.9%) patients had cutaneous involvement of varying severity. All patients had oral and ocular mucosal involvement, while 50% of patients had urogenital mucositis.

**FIGURE 1:**
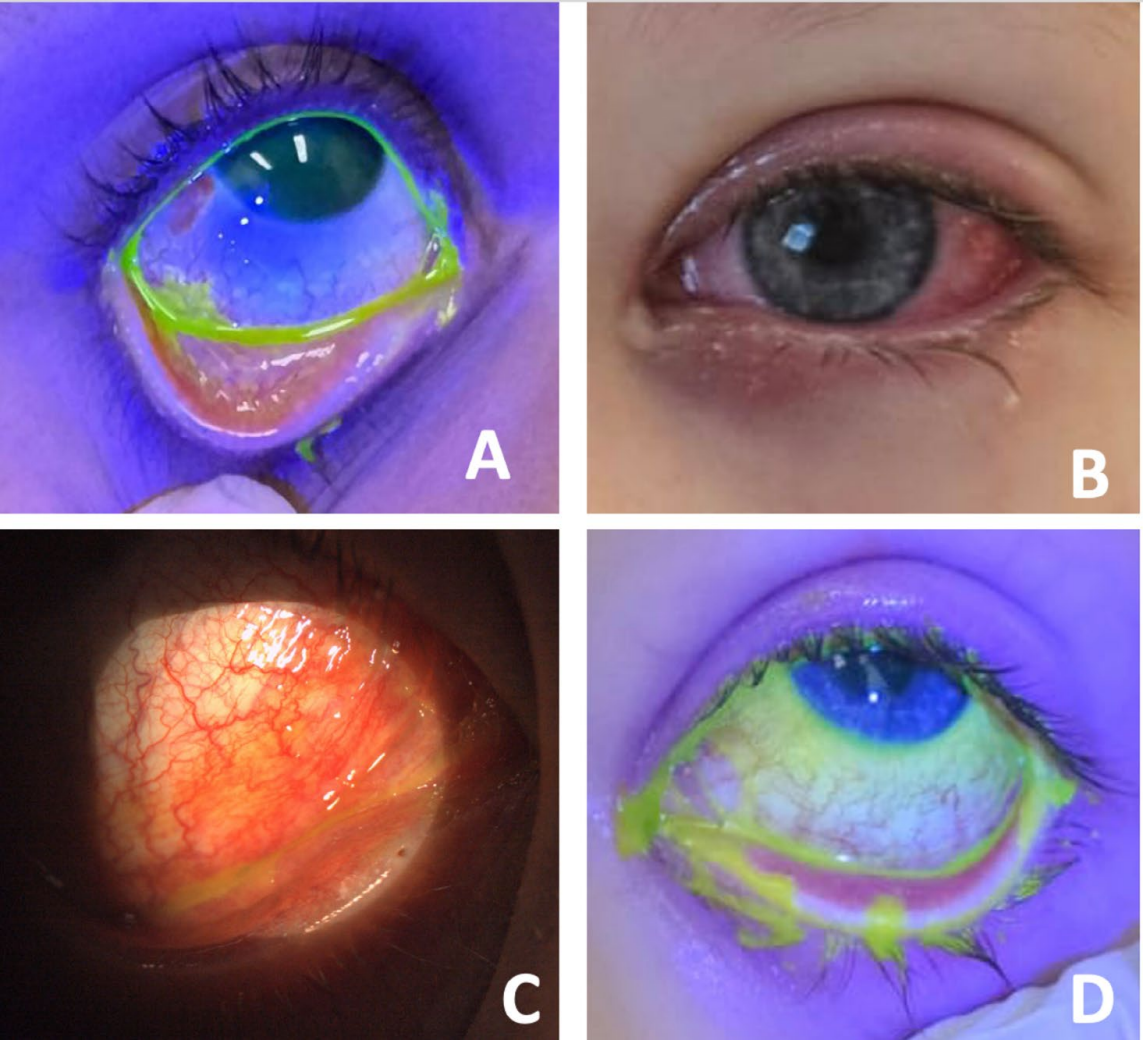
Ocular features in mild–moderate MIRM.

**FIGURE 2:**
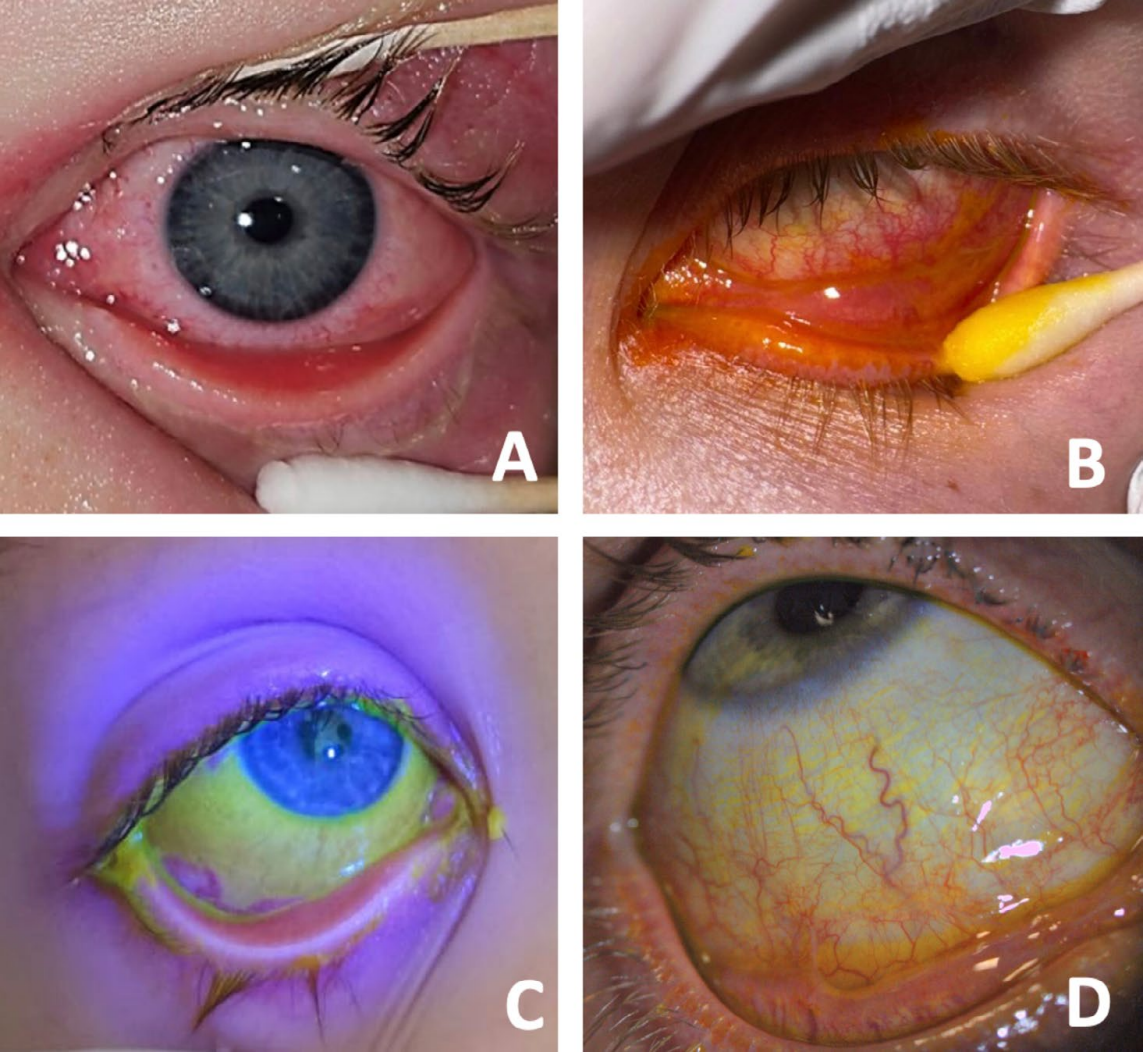
Ocular features in severe MIRM.

Ocular findings were bilateral in all cases and followed the onset of systemic symptoms by 6.2 days, with a mean duration of 23 days of ocular symptoms. At presentation, most (92.9%) patients had preserved VA with an average logMAR of 0.01 (range 0–0.2), while one patient did not have measured VA as they were intubated and ventilated at the first ophthalmological review. All patients had conjunctival injection on presentation with various degrees of severity (Figures 1 and 2). Six patients had mild–moderate ocular disease, and eight patients had severe ocular disease. Patients with severe ocular disease had a longer duration of symptoms (30.5 days) compared to those with mild–moderate ocular disease (13.23 days, *p* = 0.033).

Ocular treatment consisted of a combination of preservative‐free steroids, preservative‐free lubricants, and topical antibiotics with variable frequencies. Topical steroids prescribed were either prednisolone sodium phosphate 0.5% preservative‐free eye drops (92.9%) or hydrocortisone 1% eye ointment (7.1%) at twice‐daily to second‐hourly dosing intervals. Topical antibiotics prescribed were either chloramphenicol 1% eye ointment (78.6%) or tobramycin 0.3% eye ointment (7.1%) at twice‐daily to 4‐hourly intervals. Two patients did not receive topical antibiotics. Increase in antibiotic administration frequency was associated with shorter duration of symptoms (*p* = 0.043), whereas frequency of administration of lubricants (*p* = 0.239) or steroids (*p* = 0.066) was not significantly associated.

Pseudomembranes were debrided when identified at the bedside. One patient received an amniotic membrane graft and total glue tarsorrhaphy in one eye only, performed under general anaesthesia. This was performed due to specific clinician concern for potential severe ocular complications as seen in SJS. This patient on initial ophthalmology review had diffuse conjunctival injection and greater than 50% conjunctival surface ulceration. There was no corneal involvement or cicatricial changes. Only one eye was grafted due to patient factors, namely anxiety regarding both eyes glued closed. The amniotic membrane graft was removed after 1 week. At this time, the operated eye was found to have a corneal ED with complete resolution of conjunctival features. The fellow, unoperated eye also had complete resolution of conjunctival features at this time without cicatricial changes and no corneal involvement.

Two patients developed conjunctival complications after the resolution of their acute illness: one patient had cicatricial changes of symblepharon and forniceal shortening (Figure 1, label D). These were mild and did not require surgical intervention. The other had conjunctival discoloration and microcysts without cicatricial changes. The remainder (85.7%) of patients experienced full recovery. There was no corneal epithelial involvement for any of the patients. No patients had secondary eye infections. One patient failed to attend subsequent follow up due to self‐reported symptom resolution, the date of which was taken as the date of symptom resolution. Final VA was excellent with an average logMAR of 0.00. No patients experienced recurrence. There were no complications associated with prolonged topical corticosteroid use.

All patients received multidisciplinary care by paediatrics, ophthalmology, and dermatology teams. Systemic complications included proteinuria (7.1%), respiratory failure requiring ICU admission (21.4%), supraglottitis (7.1%) and mucosal herpes simplex virus (HSV) suprainfection (7.1%). HSV suprainfection was diagnosed based off PCR testing of affected mucosa.

## 4 Discussion

The mean age of this cohort with MIRM was 11.2 years with 75% male preponderance, which correlates with reports in existing literature [2, 5, 24] and is significantly greater than the national average of 49.3% males [25]. In this case series, 28.9% of patients with mucositis had received prior treatment with antimicrobials targeting *M. pneumoniae*. This correlates with the believed inflammatory nature of MIRM, rather than infective. Caucasians were predominantly affected in this patient cohort (78.6%), which is higher than the Australian population average of 57.6% European, American or Caucasian ancestry [26, 27]. When reported in previous studies, there was no indication that specific ethnicities were predisposed to developing MIRM [5, 8, 28]. Given the small numbers in this cohort, it is unclear if Caucasian ethnicity or male gender are risk factors for developing MIRM. Patient geographical locations were in keeping with the catchment area of the tertiary referral hospital and population density of Queensland with no clusters identified. There may have been additional cases not identified in this case series if the patients were not referred to this paediatric hospital's ophthalmology service for remote patients or patients without conjunctival mucosal involvement.

Laboratory confirmation of mycoplasma infection was found in all cases identified. Serological testing can be diagnostic if performed more than 1 week after symptom onset [29]. Serological testing in this cohort had a lower false‐negative rate compared to PCR, although this may have been confounded by PCR collection technique. Patients were often empirically treated for MIRM based on clinical diagnosis before laboratory testing confirmation. While it may be helpful to establish confirmatory testing for these patients, it is also important that treatment not be delayed while results are pending. It is therefore recommended that patients with suspected MIRM have serological testing if symptoms have persisted for over a week, with consideration of earlier PCR if required. Patients should however still commence earlier treatment based on clinical features to avoid late ocular complications.

SJS was considered as a differential for the patients who had received antibiotic therapy prior to the onset of mucositis symptoms; however, the milder clinical course, lack of corneal involvement, and cicatricial changes supported a clinical diagnosis of MIRM. It is notable, however, that the previously prescribed oral antibiotics in our patient cohort very rarely cause SJS/TEN [18, 30]. Given the overlap in clinical features of MIRM, SJS/TENS, Kawasaki disease, and erythema multiforme at presentation, it is important that the more severe conditions of SJS/TENS and Kawasaki disease be considered as a differential diagnoses in all patients presenting with mucocutaneous inflammation.

Ocular features reported in this cohort were consistent with previously reported findings—patients had milder mucosal manifestations compared to SJS/TENS [31] and had excellent visual outcomes [5, 10]. Corneal involvement has previously been reported in MIRM [6, 9, 32, 33], although none was seen in this patient cohort except for the iatrogenic corneal defect after amniotic membrane grafting. Cicatricial changes were also rarely seen (7.1%), similar to a 9.1% rate of cicatricial changes in a large systematic review [5].

None of the patients in this study had corneal involvement from MIRM, which may be a helpful clinical characteristic to aid differentiation between MIRM and SJS/TENS. The relationship between systemic and ocular severity likely reflects the underlying systemic inflammatory process and demonstrates the importance of multidisciplinary care. Early classification of ocular features may be helpful to guide systemic treatment and monitoring. For example, patients in our cohort with severe ocular disease have a higher risk of severe systemic disease and may require future respiratory support, IDC or NGT insertion and should therefore be admitted for inpatient management. It would be pertinent in future studies for general paediatrics or intensivists to stipulate systemic disease classification to more accurately assess the relationship between ocular and systemic disease severity. Systemic severity was shown to be significant as a predictive factor for days to resolution of mucositis (*p* = 0.023).

Although there was a low rate of long‐term ocular sequelae in this cohort of patients, there was a statistically significant longer symptom duration for patients classified as severe disease, compared to mild–moderate. The establishment of standardised ocular severity criteria in MIRM could guide management and long‐term follow‐up protocols for these patients. This can also be helpful for patient and parent counselling and support upon onset of their symptoms.

While antibiotic administration frequency was associated with shorter duration of symptoms, this is confounded by the fact that antibiotics were administered as ointment, potentially adding to the lubricant effect. Topical steroid administration frequency was not significantly associated with shorter ocular symptom duration; however, this may be due to small cohort numbers impacting statistical power. Overall data regarding ophthalmic therapy suggest that lower frequency of topical therapy can be just as effective as higher frequency. Lower drop frequency aids in compliance and less distress for patients and carers, particularly in a paediatric population.

Although amniotic membrane grafting has been effective in managing ocular complications of SJS/TENS and has been incorporated in other management algorithms [5, 9, 34], this was not required in this cohort. There was similar conjunctival improvement between the two eyes of the patient who received amniotic membrane grafting in only one eye; however, the grafted eye was clinically worse due to the presence of an iatrogenic corneal epithelial defect. Contrary to previous literature and management suggestions, amniotic membrane grafting did not significantly improve patient outcomes in this cohort. More data is required regarding amniotic membrane graft use in MIRM, particularly in patients with severe disease and higher risk of mucosal complications.

**FIGURE 3:**
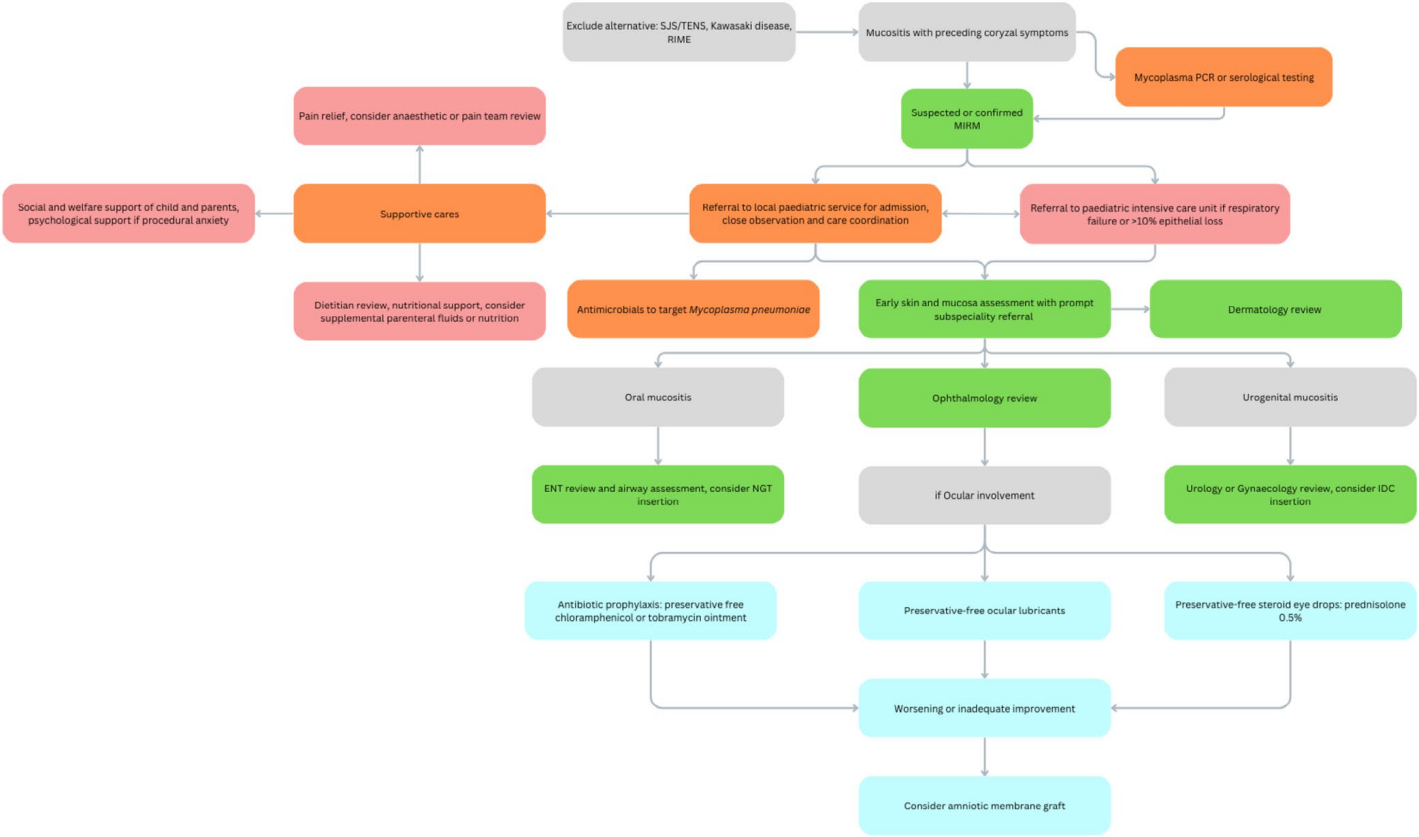
Suggested management algorithm for patients presenting with possible MIRM.

Based on the results of this study, a suggested management algorithm of paediatric patients presenting with mucositis and cough has been proposed (Figure 3). The role of systemic immunomodulators on ocular findings and outcomes is currently unknown. Corticosteroids are effective in treating pneumonia and SJS/TENS [35]. Systemic steroid or immunoglobulin therapy was not routinely prescribed in this patient cohort, and evaluation of this is beyond the scope of this paper.

## 5 Conclusion

MIRM is a relatively new clinical entity with limited literature on treatment. Given the increasing rates of *M. pneumoniae* infections, particularly in the paediatric population, the incidence of MIRM may also increase. Early ophthalmology referral and intervention improve visual outcomes and reduce the development of long‐term sequelae. The role of amniotic membrane graft in MIRM requires further research.

## Author Contributions

**Daniel Cool:** writing – original daraft, visualisation, data curation, investigation, formal analysis. **Vivien Nguyen:** writing – review and editing preparation, data curation, formal analysis. **Susan Zhang:** writing – review and editing, data curation, formal analysis. **Shiney Seo:** methodology, resources. **Shuan Dai:** visualisation, writing – review and editing, supervision, project administration.

## Conflicts of Interest

The authors declare no conflicts of interest.
